# Cathepsins as Core Players in Obesity Pathogenesis: Emerging Therapeutic Targets

**DOI:** 10.3390/biom16050730

**Published:** 2026-05-15

**Authors:** Jinghui Xie, Yingxiu Mei, Haofang Guan, Xiuwen Xia, Weijun Ding

**Affiliations:** School of Basic Medical Sciences, Chengdu University of Traditional Chinese Medicine, Chengdu 611137, China

**Keywords:** obesity, Caths, inflammation, targeted therapy

## Abstract

Obesity is a chronic metabolic disorder associated with multiple serious complications and has become a major global public health problem. Accumulating evidence indicates that members of the cathepsin (Cath) family play an important role in the development of obesity pathogenesis, thereby emerging as promising therapeutic targets for intervention. This study summarizes the multiple regulatory mechanisms of Caths involved in obesity and discusses their regulation of adipocyte differentiation, cell death, metabolism, and adipose tissue inflammation. Building on these mechanisms, we further elaborate on three novel strategies targeting Caths for obesity intervention, including selective small-molecule inhibitor development, targeted delivery systems via nanocarriers, and gene modulation approaches targeting specific Cath subtypes. Despite robust preclinical data demonstrating the efficacy of Cath-targeted interventions in ameliorating obesity and associated metabolic disorders, several critical challenges impede their clinical translation, notably: functional redundancy among Cath family members, off-target effects and unpredictable long-term safety profiles, limited subtype selectivity of existing inhibitors and immunogenicity risks associated with nanodelivery systems. To promote strategies for the clinical translation of Cath-targeted anti-obesity therapies, future research priorities should encompass artificial intelligence (AI)-driven high-throughput screening and rational design of highly selective Cath inhibitors, validation of specific Cath subtypes as clinically actionable diagnostic and prognostic biomarkers for obesity and metabolic risk stratification, and the development of personalized precision medicine strategies tailored to individual metabolic phenotypes and Cath expression profiles.

## 1. Introduction

Caths represent an evolutionarily conserved family of lysosomal proteolytic enzymes that are widely expressed in eukaryotic cells. To date, 15 Caths have been identified and classified into three main subtypes based on their catalytic mechanisms: cysteine Caths, aspartic acid Caths, and serine Caths [1]. The main function of Caths is to participate in the degradation of proteins within lysosomes. They also have an important role in physiological processes such as extracellular matrix remodeling, antigen presentation, apoptosis, and signal transduction. Caths can be used as therapeutic targets for diseases, including autoimmune diseases, cardiovascular diseases, cancer, and diabetes [2,3,4,5]. Concurrently, obesity is defined as a pervasive chronic metabolic condition characterized by excessive adipose tissue accumulation [6]. The prevalence of obesity is increasing rapidly around the world, and by itself, while not fatal, it can increase the prevalence of other diseases. It is an important factor in many diseases and their related complications. Many diseases, including cardiovascular disease, type 2 diabetes, and obstructive sleep apnea, are closely related to obesity. Therefore, the prevention and treatment of obesity is currently a major direction in medical research.

While various proteolytic systems, including matrix metalloproteinases (MMPs) and the ubiquitin–proteasome system (UPS), are implicated in obesity pathogenesis, the Cath family holds an indispensable role. MMPs primarily drive extracellular matrix (ECM) degradation to facilitate tissue remodeling, whereas the UPS predominantly regulates intracellular protein turnover and the repair of damaged DNA [7,8]. Caths, by contrast, integrate both intracellular and extracellular physiological functions. Within the cell, they process diverse hormones and growth factors and participate in autolysosomal turnover, serving as vital mechanisms for adipocyte metabolic homeostasis and lipid droplet degradation. In the extracellular space, upon secretion via lysosomal exocytosis or Golgi-mediated sorting pathways, specific Caths modulate ECM remodeling and immune cell activation by degrading matrix components and facilitating plasma membrane repair [9]. This dual functionality enables Caths to exert a highly comprehensive regulatory influence across the complex pathological network of obesity.

Existing studies have shown abnormal expression of Caths in obese patients, with some used as biomarkers of obesity. A study by Chen showed that the expression of Cath S correlated significantly with obesity-related indicators such as BMI, waist circumference, waist-to-hip ratio, and the levels of triglycerides and HDL cholesterol [10]. In addition, a recent study has further elucidated Cath L as a pivotal driver of obesity-induced chronic inflammation. Under obesity-associated stress, the expression and distribution of Cath L undergo significant change, which directly promotes obesity-associated inflammation by cleaving C3 to generate the pro-inflammatory mediator C3a [11].

Collectively, these findings corroborate that the Cath family functions as a central player in obesity pathogenesis, with substantial potential as novel targets for anti-obesity therapeutic intervention. While previous reviews have provided valuable insights into the role of Caths in metabolic diseases such as diabetes, the rapid accumulation of research findings in recent years necessitates a renewed focus on the pivotal role of Caths in metabolic diseases [12]. In particular, we must now pinpoint how these Caths participate in and regulate downstream signaling networks in obesity and critically assess their readiness for clinical translation. Therefore, this study summarizes recent research on Caths in obesity, with the aim of providing a theoretical framework for the development of innovative, Cath-based clinical therapies for obesity and its associated metabolic complications.

## 2. Methods

To provide a foundational overview of the Cath family and an in-depth analysis of their role in obesity, we conducted a narrative review by using the literature up to April 2026 in the PubMed and Google Scholar search engines. The search utilized combinations of keywords, including “cathepsin,” “obesity,” “adipose tissue,” “natural products,” “inhibitors,” and “nanocarrier delivery”. We manually curated representative, high-quality literature based on three specific thematic pillars. First, we included the key genomic and pathological studies of 15 Cath isoforms. Second, we prioritized studies explicitly elucidating the direct regulatory mechanisms and downstream signaling networks of specific Caths within the obese. Third, we integrated translational research evaluating diverse Cath-targeted interventions, specifically including natural products, endogenous protease inhibitors, synthetic inhibitors, and advanced nanodelivery systems. The present analysis highlights a focused narrative that evaluates preclinical findings regarding Cath-driven obesity and provides guidance for future clinical translation.

## 3. Caths

Caths are a distinct class of proteases that are predominantly localized within lysosomes. Although most Caths are mainly distributed in lysosomes, they can also be found in the nucleus, extracellular environment and mitochondria, indicating their diverse biological roles beyond canonical lysosomal protein degradation [13]. Caths are initially synthesized as inactive proenzymes and are glycosylated to form zymogens after translation and transported to lysosomes through vesicles [14]. Among the more than 50 hydrolases identified in lysosomes to date, Caths stand out as functionally critical components and are systematically categorized into three major classes based on their catalytic amino acid residues [15]. Of the 15 functionally validated Cath subtypes, the vast majority belong to the cysteine Caths family (11 subtypes: B, C, F, H, K, L, O, S, V, Z, W), followed by two aspartic acid Caths (D, E) and two serine Caths (A, G) [16]. To facilitate a comprehensive understanding of their structural and functional diversity, the gene locations, physiological functions and related diseases are systematically summarized in Table 1.

### 3.1. Cysteine Caths

Lysosomal cysteine Caths belong to the papain protease family, a highly conserved group of processing and digestive enzymes expressed across nearly all organisms, spanning from prokaryotes to higher eukaryotes, including humans [17]. Based on the cleavage location and peptide chain hydrolysis pattern, cysteine proteases can be divided into endopeptidases and exopeptidases [18]. Cath B is encoded by a single copy gene located on chromosome 8p22 [19], exhibiting both endopeptidase and exopeptidase activities, and is ubiquitously expressed across all human tissue types [20]. This enzyme primarily governs the selective activation of bioactive substrates such as peptide hormones and protease zymogens, as well as the degradation and remodeling of extracellular matrix (ECM) components. It is mainly involved in the development of neurological diseases, inflammatory diseases, and cancer [21,22,23]. Cath C, encoded by a gene located on chromosome 11q14.2, functions as a central upstream coordinator in immune and inflammatory signaling pathways, primarily via the activation of diverse serine proteases in immune effector cells, thereby modulating innate and adaptive immune responses [24]. The physiological functions of Cath C determine that it plays an important role in chronic inflammation, autoimmune diseases, and cancer [25,26,27]. Cath F is encoded by a gene on chromosome 11q13, close to the gene for Cath W [28]. It can mediate the antigen presentation process by degrading the major histocompatibility complex (MHC) class II-associated invariant chain and is involved in the pathogenesis of neurological diseases and cancer [29,30]. The human Cath H gene, approximately 28.5 kb long and located on chromosome 15q24-25, has a proteolytic effect in cells, thereby promoting protein homeostasis and normal cell metabolism [31]. The emerging evidence has linked abnormal Cath H activity to cancer pathogenesis, cardiovascular diseases, and neuroinflammatory disorders [32,33,34]. Cath K is encoded by a gene located on chromosome 1q21 and is mainly expressed in osteoclasts [35]. Its primary physiological function is mediating ECM remodeling by degrading collagen and it is closely related to the pathogenesis of orthopedic diseases, thyroid diseases and pulmonary fibrosis [36,37,38]. Situated on chromosome 9q21-q22, the gene encoding Cath L yields a highly active enzyme that is essential for critical biological processes, encompassing autophagy, antigen processing, and ECM remodeling [39]. Recent studies have shown its involvement in the onset of COVID-19, metastatic bone diseases, neurological diseases, and cancer [40,41,42,43]. Encoded by a locus on chromosome 4q31-q32, Cath O is involved in the turnover of proteins [44]. Currently, there are few studies on Cath O, and no association has been found between Cath O and disease. Generally, cysteine Caths exhibit optimal biological activity in acidic environments and are rapidly inactivated at a neutral pH. Unlike other members of the cysteine Caths, Cath S can remain stable and catalytically active under neutral conditions [45]. This unique biochemical property allows it to function extracellularly, making it a viable therapeutic target for the treatment of various pathological conditions. Cath S has emerged as one of the most extensively investigated members of the Cath family in disease research. It is encoded by a gene on chromosome 1q21 [46]. The hallmark function of Cath S is its role in antigen presentation. It can mediate the cleavage of the invariant chain of MHC class II, thereby promoting the presentation and migration of dendritic cells [47]. In addition, Cath S can participate in biological processes such as matrix remodeling by degrading collagen and elastin in the extracellular matrix (ECM) [48]. Existing studies have shown that Cath S is involved in the pathogenesis and progression of many diseases, including pulmonary diseases, endocrine diseases, pain, autoimmune diseases, cardiovascular diseases, nervous system diseases and cancer [49,50,51,52,53,54,55]. The gene encoding Cath V, also designated as Cath L2, is mapped to human chromosome 9q22.2 in close proximity to the Cath L locus [56]. Studies have shown that Cath V is involved in biological processes such as the turnover of elastin fibrils and the cleavage of other related substrates and is related to the occurrence of cardiovascular diseases and cancer [57,58,59]. The gene encoding Cath W is located on chromosome 11q13.1 and is mainly expressed in cytotoxic effector cells such as T lymphocytes and natural killer cells [60,61]. It processes specific substrates of cytotoxic effector cells and regulates cell-mediated cytotoxicity. It is associated with the escape of influenza virus and mediates the occurrence of influenza [62]. Cath Z, also known as Cath X, is located on human chromosome 20q13 and is widely expressed in human tissues [63]. Functionally, it modulates cellular adhesion, phagocytosis, and immune response and is involved in the development of cancer, neurological diseases, osteoporosis and aging [64,65,66,67].

### 3.2. Aspartic Acid Caths

Aspartic acid Caths, primarily comprising Cath D and Cath E, represent a small but functionally distinct subgroup of the Cath family, both of which exert pivotal regulatory roles in antigen processing and presentation [68]. As the most abundant member of this entire family, Cath D is encoded by a gene mapped to the chromosome 11p15.5 region. It is mainly involved in protein degradation, enzyme and hormone activation, antigen processing and apoptosis regulation [69]. Dysregulated expression and enzymatic activity of Cath D have been firmly implicated in the pathogenesis of diverse human diseases, including neurological disorders, endocrine metabolic diseases, and cancer [70,71,72]. In contrast, Cath E is an endopeptidase encoded by a locus on chromosome 1q32.1 [73]. Unlike the ubiquitous expression of Cath D, Cath E exhibits more tissue-restricted expression patterns, predominantly in immune cells and epithelial tissues. Its main functions include protein homeostasis, immune response regulation and cell apoptosis, and it is involved in the pathogenesis and progression of atopic dermatitis, cancer and lung diseases [74,75,76,77].

### 3.3. Serine Caths

Serine Caths mainly include Cath A and G. Cath A is an exopeptidase, with its gene localized on chromosome 20q13.12 [78]. This enzyme exhibits dual biological functions: it acts as a protective chaperone for key lysosomal enzymes, including β-galactosidase and neuraminidase, preventing their premature degradation, and simultaneously catalyzes the breakdown of various bioactive peptide hormones with high substrate specificity, such as endothelin-1 and angiotensin I [79]. It is involved in the pathogenesis and progression of cancer and cardiovascular diseases [80,81]. Cath G belongs to the neutrophil serine protease family. Its gene is located on chromosome 14q11.2 [82]. It is closely related to cardiovascular, inflammatory, and autoimmune diseases and is a key enzyme in the hydrolytic processing of receptors, enzymes, cytokines, and other bioactive peptides [83,84,85].

**Table 1:** Detailed functions of Caths and related pathologies.

Cath Type	Catalytic Type	Gene	Physiological Functions	Related Diseases	References
B	Cys	8p22-p23.1	Activates specific substrates such as hormones and zymogens and degrades the extracellular matrix	Neurological diseases, inflammatory diseases, and cancer	[19,20,21,22,23]
C	Cys	11q14.2	Activates many serine proteases in immune/inflammatory cells	Chronic inflammation, autoimmune diseases, and cancer	[24,25,26,27]
F	Cys	11q13	Mediates the antigen presentation process	Neurological diseases and cancer	[28,29,30]
H	Cys	15q24-25	Promotes protein homeostasis and normal cell metabolism	Cancer, cardiovascular diseases, and neuroinflammatory disorders	[31,32,33,34]
K	Cys	1q21	Participates in extracellular matrix remodeling	Orthopedic diseases, thyroid diseases, and pulmonary fibrosis	[35,36,37,38]
L	Cys	9q21-q22	Mediates autophagy, antigen processing and extracellular matrix remodeling	COVID-19, metastatic bone diseases, neurological diseases, and cancer	[39,40,41,42,43]
O	Cys	4q31-q32	Promotes protein homeostasis	NA	[44]
S	Cys	1q21	Cleaves MHC Class II and participates in extracellular matrix remodeling	Pulmonary diseases, endocrine diseases, pain, autoimmune diseases, cardiovascular diseases, neurological diseases and cancer	[45,46,47,48,49,50,51,52,53,54,55]
V	Cys	9q22.2	Involved in the turnover of elastin fibrils and the cleavage of other related substrates	Cardiovascular diseases and cancer	[56,57,58,59]
W	Cys	11q13.1	Involved in the processing of specific substrates of cytotoxic effector cells	Influenza	[60,61,62]
Z	Cys	20q13	Mediates cellular adhesion, phagocytosis, and immune response	Cancer, neurological diseases, osteoporosis, and aging	[63,64,65,66,67]
D	Asp	11p15.5	Involved in protein degradation, enzyme and hormone activation, antigen processing and regulation of apoptosis	Neurological diseases, cancer, and endocrine metabolic diseases	[69,70,71,72]
E	Asp	1q32	Mediates protein homeostasis, immune response regulation, and cell apoptosis	Atopic dermatitis, cancer, and lung diseases	[73,74,75,76,77]
A	Ser	20q13.12	Protects and catalyzes the degradation of enzymes	Cancer and cardiovascular diseases	[78,79,80,81]
G	Ser	14q11.2	Mediates the hydrolysis of receptors, enzymes, cytokines, and other bioactive peptides.	Cardiovascular, inflammatory, and autoimmune diseases	[82,83,84,85]

## 4. Involvement of Caths in Obesity Pathogenesis

Omics sequencing and genetic analyses have initially identified specific Caths as critical biomarkers for obesity, offering a clinical foundation for exploring their functional roles. Rather than acting as isolated mechanistic regulators, the Cath family orchestrates a continuous and interconnected pathological cascade in obesity. During the initial phase of adipose tissue expansion, specific Caths drive adipocyte differentiation by modulating ECM and adipogenic transcription factors, while concurrently regulating the cellular lipid and glucose metabolism. As adipocyte hypertrophy progresses, intracellular Caths become activated to trigger adipocyte apoptosis and oxidative stress-induced cell death, thereby compromising adipose tissue homeostasis. Within this deteriorated microenvironment, Caths propagate immune responses by cleaving key inflammatory mediators, facilitating macrophage infiltration and amplifying pro-inflammatory signaling cascades to drive obesity-associated chronic low-grade inflammation. By spanning from early adipocyte differentiation and metabolic shifts to cellular demise and systemic inflammation, distinct Cath subtypes collectively construct an interconnected pathogenic network in obesity.

### 4.1. Caths as Diagnostic Biomarkers of Obesity

The pathogenesis of obesity is highly complex and multifactorial, driven by a dynamic interplay of dietary habits, genetic predisposition, environmental influences, and socioeconomic factors. Among these contributors, genetic determinants are estimated to account for up to 70% of individual susceptibility to obesity, highlighting the critical role of genetic and molecular markers in understanding obesity etiology and identifying high-risk populations [86]. Therefore, analyzing the genetic factors that contribute to obesity may be a key approach for treating the condition. Using genomic, transcriptomic, and proteomic technologies has enabled the identification of specific Cath family members as novel genetic and circulating biomarkers for obesity, with strong correlations between Cath expression levels and key obesity-related phenotypic and metabolic traits. Taleb et al. applied high-throughput microarray technology to profile gene expression in subcutaneous adipose tissue from obese patients and lean control subjects, revealing a significant positive correlation between Cath S gene expression and obesity status and establishing Cath S as a promising novel adipose tissue biomarker for obesity [87]. Through a genome-wide association study (GWAS), Pei et al. identified Cath S as a novel susceptibility locus for obesity, reconfirming its strong genetic association with obesity [88].

**Figure 1:**
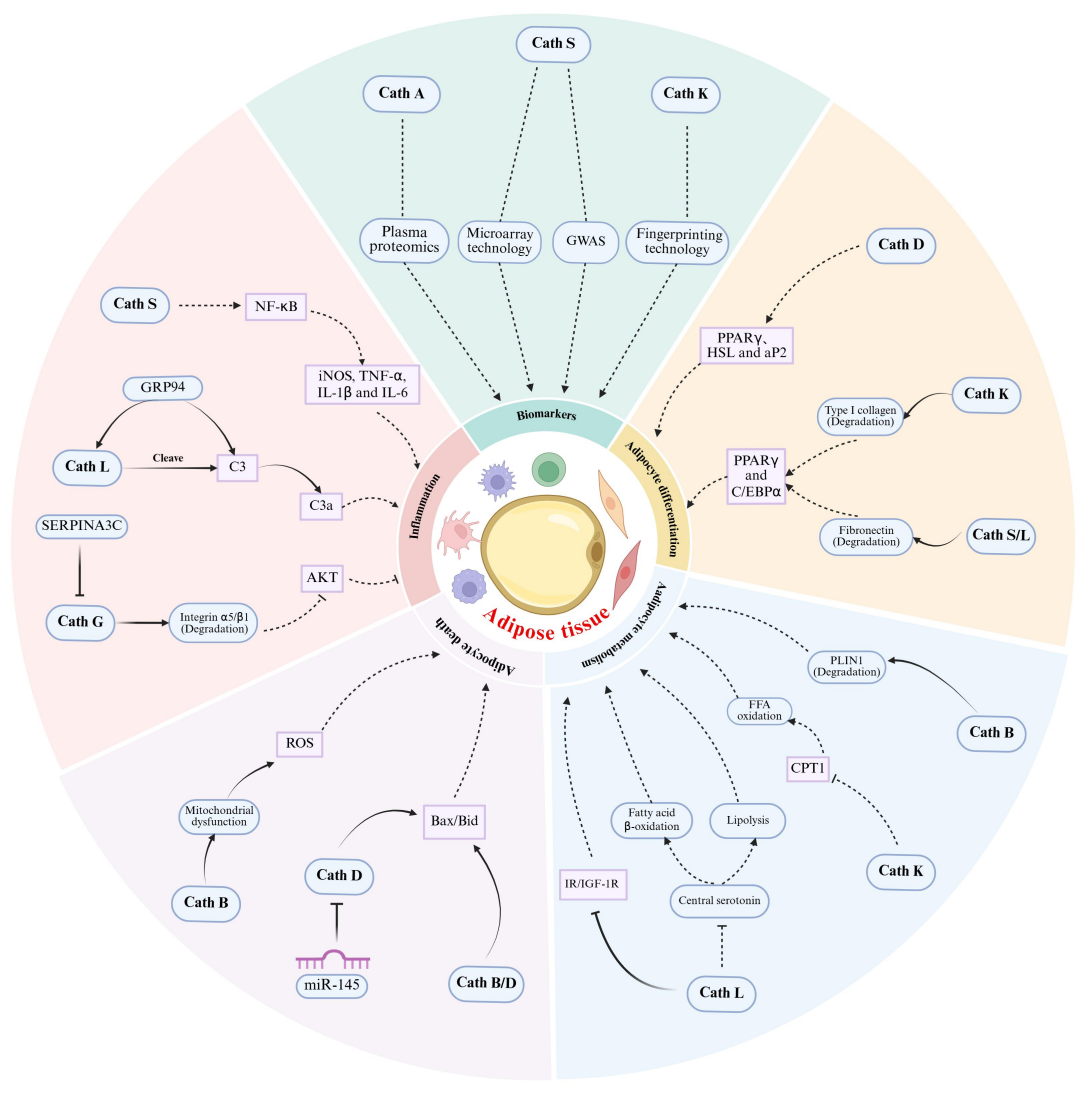
The multifaceted roles of Caths in the biology of obese adipose tissue. Caths A, S, and K serve as potential diagnostic biomarkers of obesity identified through multiomics approaches. Caths D, K, S, and L regulate adipocyte differentiation through distinct mechanisms: Cath D promotes the expression of adipogenic markers (PPARγ, HSL, and aP2), Cath K promotes the degradation of type I collagen, while Cath S and L promote the degradation of fibronectin; ultimately, both of these promote the expression of PPARγ and C/EBPα. Caths B, K, and L regulate lipid metabolism through distinct targets: Cath B modulates PLIN1, Cath K regulates CPT1, and Cath L acts upon central serotonin and the IR/IGF-1R pathways. Caths B and D promote adipocyte death: Cath B triggers mitochondrial dysfunction and subsequent ROS accumulation, and both enzymes can activate the pro-apoptotic proteins Bid and Bax. Caths S, L, and G drive adipose tissue inflammation: Cath S activates the NF-κB pathway to induce proinflammatory cytokines (iNOS, TNF-α, IL-1β, IL-6); Cath L cleaves complement C3 into C3a; and Cath G degrades integrin α5/β1 to suppress AKT signaling. In this figure, → indicates direct promotion; ⇢ indicates indirect promotion; —| indicates direct inhibition; ⋯| indicates indirect inhibition; ⋯ indicates correlative association.

Beyond Cath S, integrative multiomics studies have validated other Cath subtypes as reliable obesity biomarkers. Zaghlool et al. performed a comprehensive analysis combining large-scale plasma proteomics and Mendelian randomization, demonstrating a robust genetic link between circulating Cath A levels and obesity risk and highlighting its potential as a noninvasive circulating biomarker for metabolic risk assessment [89]. Additionally, Chiellini et al. employed low-throughput RNA fingerprinting combined with in vivo experimental validation to compare adipose tissue gene expression profiles in diet-induced obese mice and wild-type lean mice, identifying Cath K as a novel obesity-related molecular marker [90]. Collectively, these findings provide robust evidence that multiple members of the Cath family serve as reliable biomarkers for obesity, offering valuable insights into its genetic predisposition and molecular etiology. However, genetic associations do not establish these Caths as causative drivers of obesity. Validating Caths as viable therapeutic targets rather than merely associated biomarkers necessitates direct causal evidence derived from both in vivo and in vitro mechanistic investigations. These multifaceted regulatory roles, encompassing adipocyte differentiation, metabolism, cell death, and inflammation, are systematically delineated and distinguished in Figure 1.

### 4.2. Caths Regulate Adipocyte Differentiation

Mammalian adipose tissue is divided into two functional categories: white adipose tissue (WAT) and brown adipose tissue (BAT). WAT accounts for the largest proportion of total body fat localized mainly around organs and blood vessels, where it serves as a key energy storage depot and provides structural protection. In contrast, BAT accounts for a smaller proportion of total body fat and is distributed mainly in the supraclavicular, abdominal and neck areas [91]. Obesity is a metabolic state that follows the expansion of adipose tissue, with the main characteristics being accumulation of fat cells due to the enlargement of adipocytes and accumulation of fat [92]. Thus, targeted inhibition of adipose tissue differentiation may be a promising therapeutic strategy to halt or reverse obesity progression.

Accumulating preclinical evidence has demonstrated that proteases, including Caths, play pivotal roles in regulating adipocyte biological characteristics, particularly during differentiation. For instance, Masson et al. reported that Cath D expression is significantly upregulated in both the visceral and subcutaneous adipose tissue of obese humans and mice, and its expression levels gradually increase during the differentiation of preadipocytes into mature adipocytes [93]. Silencing the expression of Cath D impaired lipid accumulation and reduced the expression of biomarkers of adipocyte differentiation, such as peroxisome proliferator-activated receptor γ (PPARγ), hormone-sensitive lipase (HSL), and adipocyte fatty acid-binding protein (aP2), at the mRNA level. Han et al. demonstrated that Cath K plays a pivotal role in the early stages of adipocyte differentiation [94]. Mechanistically, Cath K promotes early adipogenic differentiation by degrading type I collagen, which subsequently induces the expression of master adipogenic transcription factors, including CCAAT/enhancer-binding protein α (C/EBPα) and PPARγ. To corroborate this, their study showed that treatment of 3T3-L1 cells with the Cath K inhibitor E-64 effectively prevented cell differentiation. Complementing these findings, Xiao and colleagues reported that Cath K inhibition prevents adipocyte differentiation, suggesting that Cath K modulates adipogenesis through ECM remodeling [95]. In addition, Caths S and L have also been implicated in adipocyte differentiation, particularly in the early stages of adipogenesis [96,97]. Specifically, these cysteine proteases facilitate preadipocyte differentiation by degrading fibronectin, which subsequently induces the expression of transcription factors C/EBPα and PPARγ. Collectively, these findings highlight the critical role of Caths in adipocyte differentiation, with mechanisms being primarily mediated through ECM remodeling and the regulation of key adipogenic transcription factors. Thus, based on current preclinical evidence, targeting Caths to inhibit adipocyte differentiation may represent a potential experimental strategy to alleviate obesity development, although its efficacy in human subjects remains to be determined.

### 4.3. Caths Regulate Adipocyte Metabolism

Adipocyte metabolic homeostasis is maintained by the precise regulation of lipid storage and decomposition and glucose uptake and utilization. Recently, Caths have emerged as key regulators of the adipocyte metabolism, with multiple subtypes implicated in the modulation of lipid and glucose metabolic pathways. For example, Mizunoe et al. showed that overexpression of Cath B regulated the adipocyte lipid metabolism by inducing ineffective basal lipolysis, specifically through the proteolytic degradation of perilipin 1 (PLIN1), a key protein that regulates lipid droplet stability and lipolysis homeostasis [98]. This degradation disrupts the lipid storage capacity and promotes abnormal lipolysis, highlighting the role of Cath B in lipid metabolic balance. Funicello et al. further confirmed the critical role of Cath K in lipid metabolism using Cath K gene knockout (KO) mice [99]. The lean phenotype observed in Cath K-deficient mice was primarily driven by a shift in the lipid metabolism, including increased mitochondrial carnitine palmitoyltransferase-1 (CPT1) activity and enhanced lipolysis, which collectively promote free fatty acid (FFA) release and oxidation rates.

Furthermore, Lin et al. demonstrated that peripheral Cath L inhibition induces fat loss through the activation of a gut–brain–adipose axis [100]. Specifically, Cath L inhibition triggers central serotonin production, which subsequently upregulates lipolysis and fatty acid β-oxidation in adipose tissue, highlighting a critical role for Cath L in systemic energy metabolism. Yang et al. further expanded on the metabolic functions of Cath L, showing that it mediates the proteolytic degradation of the β-subunits of the insulin receptor (IR) and insulin-like growth factor-1 receptor (IGF-1R) in adipocytes [97]. The inhibition of Cath L stabilizes these receptor subunits, thereby significantly enhancing the insulin-stimulated glucose uptake and improving insulin sensitivity in adipocytes. Taken together, these preclinical findings indicate that targeting Caths to regulate adipocyte lipid and glucose metabolism provides a promising anti-obesity strategy, which needs further clinical validation.

### 4.4. Caths Regulate Adipocyte Death

Beyond promoting adipocyte differentiation and lipogenesis, specific Cath subtypes actively trigger adipocyte death. For example, Eguchi showed that Caths D and B were activated in the enlarged adipose tissue or adipocytes in the epididymal adipose tissue [101]. These activated Caths induce the expression of the pro-apoptotic proteins Bax and Bid, thereby increasing adipocyte apoptosis and contributing to adipose tissue dysfunction. Kim et al. further elucidated the regulatory mechanisms underlying Cath D-mediated adipocyte apoptosis, showing that *miR-145* directly targets the 3′ untranslated region (UTR) of the Cath D gene, negatively regulating its expression at the post-transcriptional level and consequently inhibiting adipocyte death [102]. Gornicka et al. reported that FFA treatment induces lysosomal permeability in adipocytes, leading to the release of Cath B into the cytoplasm. Cytoplasmic Cath B directly induces mitochondrial dysfunction, promoting reactive oxygen species (ROS) generation and subsequent adipocyte death [103]. Notably, in contrast to its role in promoting adipocyte differentiation, Cath D also induces adipocyte death through distinct biological pathways. This dual regulatory function of Cath D on adipocytes may depend on cell-specific threshold effects, tissue microenvironment signals, or expression levels. Ultimately, these studies consistently confirm that inhibiting Cath D expression or activity is beneficial for regulating obesity, as it both suppresses adipocyte differentiation and reduces excessive adipocyte death, thereby maintaining adipose tissue homeostasis.

### 4.5. Caths Regulate Obesity-Associated Inflammation

Obesity has been recognized as a key driver of systemic chronic low-grade inflammation, characterized by a vicious cycle of immune cell infiltration into adipose tissue, the release of proinflammatory cytokines, and the development of metabolic disorders—all of which contribute to obesity progression and its comorbidities [104]. Recent studies have identified Caths as central players in obesity-mediated inflammatory signaling. For example, Li et al. reported that SERPINA3C, a serine protease inhibitor, prevented the turnover of integrin α5 and integrin β1 proteins in adipocytes by inhibiting Cath G activity, thereby promoting AKT activity and inhibiting inflammation [105]. Wang et al. confirmed that the endoplasmic reticulum stress protein glucose-regulated protein 94 (GRP94) promoted the secretion of Cath L and complement C3 by palmitic acid (PA)-stressed M_2_ macrophages [11]. Complement C3 and its cleavage fragment C3a are important participants in obesity-related low-grade inflammation [106]. Further supporting the clinical relevance of these mechanistic findings, a clinical cross-sectional study showed that the expression of Cath L in the omental adipose tissue of obese patients is positively correlated with the expression of three proinflammatory factors, C-C motif chemokine ligand 2 (CCL-2), interleukin-6 (IL-6), and interleukin-1β (IL-1β), suggesting a potential role for Cath L in driving inflammation in human visceral adipose tissue [107]. Furthermore, Zheng et al. provided in vivo evidence that inhibiting Cath S activity suppresses the inflammatory response in obese mice [108]. Specifically, a Cath S inhibitor curtailed macrophage recruitment to adipose tissue and downregulated the release of key inflammatory mediators—including inducible nitric oxide synthase (iNOS), tumor necrosis factor-α (TNF-α), IL-1β, and IL-6—by inhibiting the nuclear factor-κB (NF-κB) signaling pathway in adipose tissue. The above studies thus indicate that targeted inhibition of Caths may be a potential strategy to alleviate obesity-associated inflammation and improve metabolic outcomes. Although current preclinical studies have delineated the specific pathways through which individual Caths regulate obesity pathology, these findings warrant cautious interpretation. Because members of the Cath family exhibit extensive functional redundancy and synergistic effects, evaluating a single member in isolation may fail to fully capture the complex, coordinated regulatory networks within the obese adipose tissue microenvironment. Elucidating these intricate synergistic and compensatory dynamics represents a critical step toward assessing the potential of single-target therapies and advancing the development of targeted anti-obesity drugs.

## 5. Potential Therapeutic Approaches Targeting Caths

Building on the multifaceted regulatory roles of Caths in obesity pathogenesis, robust human clinical data, including the genome-wide association studies and large-scale plasma proteomics discussed in Section 4.1, have firmly established them as critical biomarkers in human obesity. Integrating these human clinical data with obesity pathological mechanisms strongly indicates the potential of Caths as translatable therapeutic targets. Consequently, substantial progress has recently been made in the preclinical development of Cath-targeted therapeutic approaches, encompassing natural products, endogenous and synthetic Cath inhibitors, and nanocarrier-mediated targeted delivery systems. These preclinical strategies aim to selectively modulate Cath activity, overcome pharmacological limitations such as off-target effects and poor bioavailability, and ultimately ameliorate obesity. However, the current limitation is that while human multi-omics data supports the pathological relevance of Caths, human clinical trial data evaluating Cath-targeted interventions specifically for obesity management remains scarce. Future translational efforts must address this limitation by transitioning from human biomarker identification and preclinical inhibitor successes to rigorous clinical therapeutic validation. In the following sections, we will evaluate the diverse pharmacological strategies that are currently being explored to address this translational gap, encompassing natural products, endogenous and synthetic Cath inhibitors, and nanocarrier-mediated targeted delivery systems.

### 5.1. Natural Products

Natural products derived from plants, animals and microorganisms have gained increasing attention in disease intervention and treatment due to their inherent advantages, including low toxicity, multitarget action, and good biocompatibility. Accumulating preclinical evidence has demonstrated that several natural products can target Caths to regulate obesity onset and progression, providing a promising avenue for the development of safe and effective anti-obesity agents. In this regard, a study by Xie et al. demonstrated that the isoquinoline alkaloid liensinine extracted from lotus seeds inhibited the activity of corresponding Caths by promoting the accumulation of proteases B, D, and L [109]. This accumulation inhibited mitochondrial autophagy and prevented the conversion of beige fat to white fat cells, thereby preventing the occurrence of obesity. In vitro cellular assays confirmed that a quantitative working concentration of 10–40 µM dose-dependently promoted the accumulation of Cath pro-forms, while toxicological assessments established a strict cytotoxicity boundary at 60 µM. Another study using proteomics to investigate the regulatory effects of *Ginkgo biloba* extract (500 mg/kg/day) on oxidative stress in obesity showed that ginkgo extract upregulates Cath D expression [110]. This upregulation may induce adipocyte apoptosis and reduce the number of hypertrophic adipocytes, while the extract’s inherent potent antioxidant properties simultaneously alleviate oxidative stress. Although the oral dosage demonstrated favorable metabolic improvements during a 14-day treatment without affecting appetite, the study did not quantitatively evaluate its systemic pharmacokinetic parameters or long-term toxicological safety. Myoung et al. probed the antiadipogenic properties of the water extract of *Paecilomyces tenuipes* (PTW), an entomopathogenic fungus [111]. They demonstrated that PTW exhibits dose-dependent inhibition of Cath S activity, thereby suppressing Cath S-induced adipocyte differentiation and lipid accumulation. Approximately 250 μg/mL of PTW can reduce Cath S activity by half. In 3T3-L1 preadipocytes, 200 μg/mL of PTW treatment for 3 h reduced intracellular Cath S activity by 73.0%. In vivo supplementation with PTW (1 g/kg body weight/day for 8 weeks) reduced body weight gain and adipose tissue expansion in HFD-fed mice, highlighting PTW as a promising natural Cath S inhibitor in preclinical obesity models, laying the foundation for future clinical translation studies. These preclinical studies underscore the potential of natural products as novel Cath-targeted agents for obesity treatment.

### 5.2. Synthetic and Endogenous Cath Inhibitors

#### 5.2.1. Endogenous Protease Inhibitors

Endogenous Cath inhibitors are the “molecular brake” of Caths and participate in the regulation of diseases by precisely tuning the activity of Cath proteases. Currently, endogenous Cath inhibitors are divided into two major categories: serine protease inhibitors and cysteine protease inhibitors. Among these, the serpin superfamily is the largest group of protease inhibitors in nature, comprising 16 evolutionary branches (A-P) [112]. Serpins interact with Caths in two distinct ways: an inhibitory pathway (directly blocking Cath activity) and a substrate pathway (acting as a decoy substrate to divert Cath activity) [113]. Li et al. revealed that SERPINA3C, a secreted member of the serpin A branch, reduced inflammation in obese adipose tissue by inhibiting the Cath G/integrin/AKT pathway, highlighting the potential of endogenous serpins as therapeutic targets for obesity-associated inflammation [105]. Beyond obesity, Burgener et al. demonstrated the critical role of endogenous protease inhibitors in immune cell homeostasis: Serpinb1a and Serpinb6a promote the survival of monocytes and neutrophils by inhibiting Cath G activity [83]. This inhibition prevents Cath G-mediated cleavage of gasdermin D (GSDMD), thereby blocking programmed necrosis and reducing GSDMD-driven inflammation. Serpinb6 and Serpinb1 inhibit Cath G with exceptionally rapid second-order rate constants of 10^7^ mol/L^−1^·s^−1^ and 2 × 10^6^ mol/L^−1^·s^−1^, respectively. While Serpinb1 exhibits a broad inhibitory profile against multiple neutrophil serine proteases, including CatG, neutrophil elastase, and proteinase-3, Serpinb6 displays a highly restricted specificity, exclusively targeting Cath G. Furthermore, Lo et al. reported that Serpin B13 restricts the proliferation of insulin-secreting β-cells by inhibiting Cath L [114]. Enhancing Cath L activity by blocking Serpin B13 was shown to promote β-cell expansion via the modulation of E-cadherin signaling. These findings suggest that investigating the action and effects of more endogenous Cath inhibitors, particularly those targeting Caths G and L, may provide a novel approach for obesity.

#### 5.2.2. Synthetic Cath Inhibitors

Exogenous synthetic Cath inhibitors have become a research hotspot in Cath-targeted therapy, owing to their high specificity and controllable pharmacological intervention. In the past two decades, many patents have been applied for synthetic inhibitors that target Caths and have revealed that these inhibitors are an effective means of treating various Cath-related diseases [115,116]. Preclinical studies highlight RO5444101 as a highly specific Cath S inhibitor that directly targets the active site with an exceptional inhibitory constant of 0.13 nmol/L [117]. In vitro studies have confirmed that the 50% inhibitory concentration (IC50) of RO5444101 against mouse Cath S is 0.3 nmol/L, whereas its IC50 against human Cath K, Cath L, Cath C, Cath X, and Cath H exceeds 25,000 nmol/L, thereby demonstrating its high specificity. In cellular assays, RO5444101 effectively blocks the cleavage of the major histocompatibility complex class II-associated invariant chain, leading to the accumulation of the invariant chain-p10 fragment with a mean half-maximal effective concentration of 189.3 nmol/L. Following a 12-week administration at 60 mg/kg in mice, RO5444101 significantly suppressed high-fat diet-induced adipogenesis, inflammatory infiltration, and hepatic lipid accumulation; notably, no macroscopic toxicological signs, such as peritonitis, pain, or discomfort, were observed upon necropsy [108]. Furthermore, rather than inducing hepatotoxicity, RO5444101 significantly attenuated the abnormal elevations of serum aspartate aminotransferase and alanine aminotransferase driven by the high-fat diet. These findings demonstrate that RO5444101 poses no significant hepatotoxic risk. Similarly, Mizunoe et al. used a Cath B-specific rapid inactivator, CA074ME, to improve the metabolic function of adipocytes [98]. CA074ME is a cell-permeable, highly specific proinhibitor whose active free acid form, CA074, irreversibly inactivates Cath B through covalent binding between its epoxide group and the Cys29 residue (with a reaction rate constant reaching as high as 112,000 M^−1^·s^−1^). Due to its negative charge, free CA074 suffers from poor membrane permeability. To overcome this, researchers developed the electroneutral prodrug CA074ME by methyl esterifying the carboxyl group of its terminal proline. While CA074ME readily penetrates cell membranes, its intrinsic inactivation rate against Cath B is reduced by over three orders of magnitude to 68 M^−1^·s^−1^. Upon cellular entry, CA074ME undergoes deesterification, releasing the potent CA074; this mechanism achieves 95% specific inactivation of intracellular Cath B in human gingival fibroblasts within 3 h at 1 μM [118]. In cellular pharmacological models, CA074ME exhibited a favorable in vitro safety profile; continuous 24 h incubation at concentrations up to 10 µM neither induced cytotoxicity nor compromised cell viability. Another in vivo study demonstrated that when CA074Me was continuously infused into the brains of guinea pigs via osmotic micropumps at a dose of 0.15 mg/kg/day for 7 and 30 days, the animals remained healthy, exhibited no signs of toxicity, and displayed normal behavior [119]. Yang et al. demonstrated that CLIK195—a specific inhibitor from this series—effectively suppresses adipocyte differentiation and ameliorates obesity by targeting Cath L [97]. The CLIK195 achieves high subtype selectivity through the introduction of a rigid, bulky phenyl ring adjacent to the amino group of the common epoxysuccinate backbone, allowing the backbone to irreversibly bind to and inactivate the enzyme’s active site. Enzymatic assays demonstrated its exceptional target specificity: at a concentration of 10^−6^ M, the CLIK195 completely inhibited Cath L while exhibiting no inhibitory effect against other family members, including Caths B, K, and C. It demonstrates robust metabolic stability, retaining 100% of its parent activity after a 1 h incubation in metabolic enzyme-rich tissue homogenates. Cellular assays indicated that CLIK195 at 10^−6^ M achieved 50% inhibition of Cath L activity in splenocytes. Murine experiments confirmed that intraperitoneal doses ranging from 1 to 10 mg/kg successfully penetrated hepatic lysosomes, producing a definitive, dose-dependent inhibition of Cath L. Importantly, CLIK195 possesses a highly reliable toxicological and safety profile. In vitro, effective working concentrations up to 10 µM induced neither cytotoxicity nor a decline in cell viability. In vivo, long-term intraperitoneal administration at high doses up to 100 mg/kg/day caused no significant alterations in the core physiological and energy expenditure parameters, including the total food and water intake, urinary and fecal excretion, and basal metabolic rate. Xiao et al. used the Cath K inhibitor E-64 to inhibit the activity of Cath K in adipocytes and regulate adipocyte differentiation [95]. E-64, an irreversible and broad-spectrum cysteine protease inhibitor, exhibits extremely high selectivity for cysteine proteases; it effectively inhibits Caths B, H, and L, yet remains completely ineffective against serine proteases or metalloproteases, even at concentrations as high as 0.5 mM [120]. The isobutyl group of E-64 deeply embeds into the S2 hydrophobic binding pocket of Cath K, while its guanidinium group forms stable interactions with residues within the S3 pocket. Nucleophilic attack by Cys25 on the epoxide ring of E-64 triggers ring opening, resulting in the formation of a stable covalent thioether bond between the sulfur atom of Cys25 and the C2 atom of E-64. This stable binding permanently occupies the active site, leading to the loss of enzymatic activity [121]. In cellular pharmacological models of obesity and adipogenesis, E-64 (0–5.0 µM) dose-dependently inhibited the differentiation of 3T3-L1 preadipocytes into mature adipocytes. Notably, safety and cytotoxicity assessments revealed that both the Trypan Blue exclusion test and microscopic observations confirmed the absence of any cytotoxic signs, demonstrating its safety profile at the cellular level. These synthetic inhibitors demonstrate good targeted pharmacological efficacy and preliminary safety profiles in preclinical models of obesity. Meanwhile, considering the clinical relevance of Cath S as a potential biomarker in human obesity cohorts, the development of highly effective inhibitors such as RO5444101 is of great significance for the clinical translation of Cath-targeted therapy for obesity. Despite the favorable outcomes of these Cath inhibitors in preclinical models, the inherent metabolic and physiological differences between mice and humans warrant cautious interpretation of these findings. Moreover, the potential toxicity associated with synthetic inhibitors constitutes a formidable barrier to their clinical translation, particularly the adverse effects arising from systemic non-specific inhibition and off-target interactions. Addressing these translational gaps and facilitating the clinical application of these drug candidates will necessitate the development of precision-designed inhibitors and targeted nanodelivery systems, alongside comprehensive human pharmacokinetic studies and long-term clinical toxicological assessments.

### 5.3. Targeted Delivery by Nanocarriers

In recent years, targeted inhibitors of Caths have been studied extensively, and patents have been applied for obesity. However, considering that the physical and chemical properties of small molecule drugs themselves will affect their absorption and conversion in the body, there is an urgent need to prevent these changes and improve the therapeutic performance of drugs. To overcome these limitations, nanocarrier systems have emerged as a powerful technology to modify the pharmacokinetic characteristics of drugs, improve their bioavailability, and achieve targeted delivery to specific tissues or cells, thereby enhancing therapeutic efficacy and reducing off-target effects. Currently, numerous researchers are constructing nanodelivery systems for Cath inhibitors to optimize their therapeutic performance. For example, Hassan et al. constructed a chitosan-coated nanostructured lipid carrier (NLC) to deliver tanshinone IIA, a natural compound that inhibits Cath B, for the treatment of Parkinson’s disease [122]. Chitosan coating imparts a positive surface charge to the nanostructured lipid carriers, facilitating mucoadhesion and enhancing intranasal drug retention. Moreover, the modified nanocarriers attenuate the rapid burst release profile, decreasing the 24 h release rate from the 80% observed with the free drug to 55%. In vivo evaluations demonstrate that intranasal administration at a low dose of 0.3 mg/kg effectively bypasses the blood–brain barrier to target the brain. Compared to their uncoated counterparts, these chitosan-modified nanocarriers achieved an additional 28.4% reduction in striatal Cath B expression, demonstrating superior targeted inhibitory efficacy and improved drug utilization. Kozak et al. developed liposomes functionalized with Pepstatin A, a potent natural inhibitor possessing a highly specific binding affinity for Cath D (dissociation constant ≈ 0.4 nM), with the resulting carriers exhibiting an average diameter of 94 nm and a PDI of 0.1 [123]. In vitro evaluations revealed that, compared to non-functionalized liposomes, these Pepstatin A-conjugated lipid carriers achieved significantly greater accumulation on the surface of breast cancer cells, thereby enhancing the anti-proliferative efficacy of co-delivered chemotherapeutics. Mikhaylov et al. developed nanosized stealth liposomes (average diameter approximately 79.2 nm) integrating the Cath B inhibitor NS-629 [124]. Active-site titrations revealed that this lipidated inhibitor binds the catalytic center of recombinant Cath B with an apparent 1:1.4 stoichiometry, ensuring highly specific and irreversible enzyme inactivation. Cytotoxicity assays demonstrated that loading the chemotherapeutic doxorubicin into these targeted nanocarriers increased its potency against breast cancer cells by 22-fold compared to non-targeted liposomes, drastically reducing the IC50 from 1.1 μg mL^−1^ to 0.05 μg mL^−1^. In addition, Yang et al. engineered a dual-inhibitor nanocarrier (approximately 100 nm in diameter) loaded with the Cath L inhibitor E-64d and the TMPRSS2 inhibitor camostat [125]. It exhibited highly controllable drug release kinetics, effectively preventing premature leakage at a physiological pH of 7.4 while demonstrating rapid, pH-dependent release in simulated acidic lysosomal environments. By binding to intact erythrocytes via hydrogen bonds or electrostatic interactions, these carriers formed “nanoengineered Red Blood Cells” that directly reached the lungs upon intravenous injection, thereby enabling the drug-loaded nanocomposites to evade macrophage-mediated phagocytosis in the liver and spleen. Consequently, this composite carrier achieved precise targeting of the essential proteases and blocked the pulmonary invasion of severe acute respiratory syndrome coronavirus 2 (SARS-CoV-2). Pathological analyses of major organs revealed no discernible lesions in the treated group, confirming the absence of significant organ toxicity. Advanced delivery systems are designed to minimize the systemic off-target effects, a key pathological insight derived from human clinical multi-omics data. In this context, nanocarrier-mediated delivery provides a promising solution, significantly enhancing targeted tissue penetration and improving the bioavailability of small-molecule inhibitors. The clinical translation remains constrained by critical biosafety limitations, as material-induced immunogenicity and unpredictable long-term accumulation in vital organs pose substantial safety risks. Ensuring the safe advancement of these delivery materials into human clinical trials will necessitate rigorous pharmacokinetic and toxicological evaluations.

## 6. Future Research and Prospects

### 6.1. State of the Art and Current Knowledge Gaps

The state of the art in this field has established the critical regulatory role of Caths in the pathogenesis of obesity. The existing studies have successfully indicated the core downstream signaling networks governed by Caths in obesity. Preclinical studies demonstrate that targeting specific Cath subtypes drives profound anti-obesity and metabolic improvements. These important findings represent the current pinnacle of research into Cath-targeted therapies for obesity. Despite this series of remarkable progress, a comparative comparison between the state of the art and current clinical needs reveals that numerous issues remain that urgently require resolution. These issues impede the translation of such technologies into clinical practice.

First, the gap in synergistic networks. The majority of existing studies have focused on the individual functions of single Cath subtypes. However, the Cath family exhibits extensive functional crosstalk and synergistic or compensatory regulatory effects on adipocyte biology, extracellular matrix remodeling, and adipose tissue inflammation. Currently, there is a lack of systematic, comprehensive research exploring the collective and interactive effects of multiple Caths, and the critical gap of whether this functional redundancy and synergism contribute to the limited efficacy of single-target Cath inhibitors observed in preclinical and preliminary clinical settings remains.

Second, the gap in translational models. While state-of-the-art interventions demonstrate potent efficacy in rodent models, species differences in physiology, metabolism, and Cath expression patterns between rodents and humans raise substantial uncertainty. A major gap in clinical translation remains; specifically, whether these inhibitors can achieve comparable therapeutic efficacy, metabolic improvement, and safety in obese patients remains unvalidated. Therefore, large-scale, well-designed clinical trials are required to validate their clinical value.

Third, the gap in toxicological safety. While short-term preclinical models demonstrate the therapeutic potential of Cath inhibitors in obesity, underlying systemic toxicity remains a major barrier hindering clinical translation. Given the essential physiological roles of the Cath family across multiple organs, non-specific pan-inhibition poses severe safety risks, including collateral organ toxicity. A cautionary precedent for this translational challenge is the Cath K inhibitor odanacatib, which was ultimately terminated during Phase III clinical trials for osteoporosis due to severe cardiovascular toxicity [126]. Compounding this issue, the highly conserved active site architecture among Cath family members often leads to off-target toxicity caused by insufficient inhibitor selectivity. Guaranteeing the safety of targeted Cath inhibitors in chronic obesity management will necessitate the urgent development of precision-designed inhibitors and organ-specific targeted delivery systems.

Fourth, the gap in delivery biosafety. Although the nanocarrier-mediated targeted delivery of Cath inhibitors represents great promise in overcoming the limitations of small-molecule drugs, the biosafety of these systems remains a major obstacle. A key concern associated with lipid and polymeric nanocarriers is their potential immunogenicity, which can trigger innate or adaptive immune responses, reduce drug stability, and cause adverse systemic effects [127]. Additionally, the long-term biocompatibility, biodegradation pathways, and potential accumulation of nanocarriers in vital organs remain insufficiently characterized, warranting in-depth safety assessments before clinical application.

### 6.2. Future Research Directions

Since ancient times, natural secondary metabolites derived from plants, bacteria and fungi have provided a good source of drugs, owing to their structural diversity, low toxicity, and multitarget biological activities. Accordingly, the development of novel natural product-based Cath inhibitors represents a promising strategy for safe and effective obesity intervention. Future research should prioritize the integration of AI technologies to streamline inhibitor development, rationally design dual- or multi-target Cath inhibitors, and optimize pharmacological properties.

Currently, clinical investigations of Cath-targeted therapies for obesity remain scarce, with most studies confined to the preclinical stage. A critical future direction is to accelerate the translation of promising preclinical candidates into rigorous phase I, II, and III clinical trials, systematically evaluating their efficacy, safety, pharmacokinetics, and long-term tolerability in obese human subjects. Furthermore, the validation of Caths as clinically actionable biomarkers—such as circulating Cath S levels—can be integrated into clinical practice for early obesity screening, metabolic risk stratification, and therapeutic response monitoring, enabling precise and personalized intervention.

Beyond conventional drug development, precision medicine strategies tailored to individual genetic backgrounds, Cath expression profiles and metabolic phenotypes represent a cutting-edge direction for obesity treatment. Developing personalized therapeutic regimens based on patient-specific Cath gene polymorphisms and expression patterns can maximize the therapeutic efficacy while minimizing the off-target effects. Additionally, exploring combination therapies targeting multiple Cath subtypes or combining Cath inhibitors with existing anti-obesity interventions may overcome functional redundancy and achieve synergistic metabolic benefits.

## 7. Conclusions

The Cath family functions as a pivotal regulator of obesity pathogenesis by regulating adipocyte differentiation and maturation, programmed adipocyte death, lipid and glucose metabolism, and chronic low-grade inflammation within adipose tissue. While targeting Caths has shown highly promising results in preclinical models as an innovative strategy for treating obesity, the translational gap to clinical application remains a critical challenge. Future research must conduct well-designed clinical trials to evaluate whether these preclinical research results can be safely and effectively extrapolated to human patients. Nevertheless, substantial challenges—including functional redundancy, limited clinical data, and nanocarrier biosafety concerns—must be addressed to facilitate a successful clinical translation.

## Data Availability

Not applicable.
